# Quantitative Analysis of GABA_A_ Gamma Receptor Subunits in the Developing Embryonic Chick Forebrain

**Published:** 2012

**Authors:** Sayed Rasul Zaker, Abolghasem Esmaeili, Majid Bouzari, Elham Shirani

**Affiliations:** 1 *Department of Biology, Faculty of Sciences, University of Isfahan, Isfahan, Iran*; 2 *Department of Biotechnology, Faculty of Advanced Sciences and Technologies, University of Isfahan, Isfahan, Iran*

**Keywords:** Developmental expression, Embryonic forebrain, GABAA Receptor subunit, Real-time RT-PCR

## Abstract

**Objective(s):**

In this study we investigated the expression of GABA_A_ receptor subunits during brain development. These receptors may change in the embryonic chick forebrain.

**Materials and Methodes:**

The expression levels of four types of GABA_A_ receptor gamma subunits (γ1, γ2, γ3 and γ4) were quantified in the embryonic chick forebrain at 32 hr, 3, 7, 14, and 20 days of incubation and day one after hatching. The expression level of mRNA in the forebrain of embryonic chicken was measured using real-time RT-PCR.

**Results:**

The expression level of each subunit increased gradually with development and reached a plateau on 20th day of embryonic development. A reduction was observed on day one after hatching in all gamma subunits.

**Conclusion:**

This may explain the different physiological and pharmacological function of GAB_A_ receptor gamma subunits before and after hatching.

## Introduction

Gamma-aminobutyric-acid (GABA) is a major inhibitory neurotransmitter in the centeral nervous system (1, 2) that intervenes most of its effects through GABA_A_ receptors. GABA receptors are classified into two major groups: ionotropic GABA type-A receptors that form ion channels (GABA_A_Rs) and metabotropic GABA type-B (GABA_B_Rs) which are G protein-coupled receptors (3). The GABA_A_R is a member of the cysteine-loop family of ligand-gated ion channels. Receptors of this class comprise five subunits arranged symmetrically around a central ion-conducting pore. Each subunit consists of four α-helical transmembrane domains and a large extracellular amino-terminal domain that harbors the ligand binding sites and the signature cysteine-loop (4).

The channel formed by these receptors, is a pentameric assembly resulting from five of at least 21 subunits, grouped in the eight classes: alpha (α1-6), beta (β1-4), gamma (γ1-4), delta, pi, epsilon, theta, and rho (ρ1-3) (2, 3) which permits an immense number of putative receptor isoforms.

The subunit combination of a GABA_A_Rs determines not only their natural functions but also the specific effects of allosterical modulators of benzodiazepines, barbiturates, steroids, general anaesthetics, some convulsants, polyvalent cations, and ethanol (5-9). 

GABA_A_Rs may have different physiological and pharmacological functions based on their expression in different stages of the chicken life (10-12). Since quantitative changes in GABA_A_ receptor gamma subunits during embryonic development are not well-known, the aim of present study was to demonstrate the presence of these subunits expression in chicken forebrain.

## Materials and Methods

***Animals***

Fertile eggs of Ross breed obtained from a comercial parent farm were used in all experiments. Efforts were made to minimize animal suffering and the number of animals used. All experiments were approved by the Animal Ethics Committee for the University of Isfahan, Isfahan, Iran.

***Preparation and dissections***

The fertilized eggs were incubated in a standard incubator (Gujiran, Iran) at standard conditions. The embryo were collected at the specified times (32 hr, 3, 7, 14, 20, and 21 days) and the forebrain were dissected under a binocular microscope (Olympus). At the early stages of development, whole brain was dissected. 

***Tissue preparation***

At specified times (32 hr, 3, 7, 14, 20, and 21 days after incubation), the whole brain at 33 hr after hatching and forebrain of the animals were rapidly removed from the fertelized eggs (n= 12 for each time point) and placed into ice-cold RLT (Qiagen). The tissue segments were stored at -70 ^°^C.

***RNA extraction and RT***

Total cellular RNA was isolated from frozen tissues using the RNeasy Plus Mini Kit (Qiagen) for isolation. The extracted RNA was dissolved in diethyl pyrocarbonate (DEPC) treated water. The purity and integrity of the extracted RNA was evaluated by optical density measurements (260/280 nm ratios) and by visual observation of samples on agarose gel electrophoresis. Both methods indicated integrity of the extracted RNA with little or no protein contamination. Complementary DNA synthesis reactions were performed using 1 µg DNase (Fermentas) which treated total RNA from each sample and cDNA Synthesis Kit (Fermentas) with random hexamer (Fermentas) priming in a 20 µl reaction according to the manufacturer’s instructions.

***Quantitative RT-PCR***

Real-time PCR was performed in the Chromo4 Detection System (BioRad, USA). Briefly, 20 ng of cDNA and gene specific primers were added to SYBR Green Master Kit (Takara, Tokyo, Japan) and subjected to PCR amplification (1 cycle at 95 ^°^C for 30 sec, and 45 cycles at94 ^°^C for 5 sec, 55 ^°^C for 20 sec, and 72 ^°^C for 34 sec). All PCR reactions were run in duplicate. The amplified transcripts were quantified using the comparative Ct method (http://www.pebiodocs.com/pebiodocs/04303859.pdf.). 

**Figure 1:**
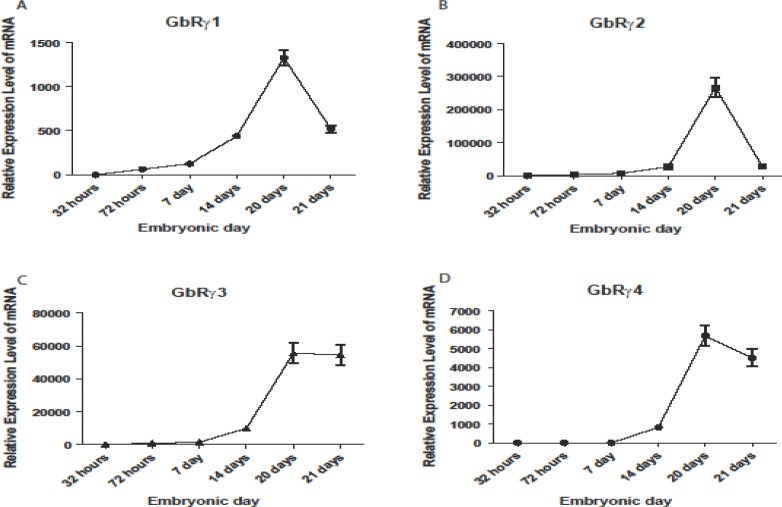
Quantitative RT-PCR analysis of GABA_A_ receptor gamma 1 (A), gamma 2 (B), gamma 3 (C), and gamma 4 (D) mRNA expression during chick embryonic development. Expression of the investigated genes simultaneously elevated on day 20. Expression pattern of all of these genes was different. Error bars show mean±SD

Gene-specific primers were designed using Beacon Designer 7.5 software. The primers used for real-time PCR were:

Gbrγ1: 5'- AGGACATCTCTGGGTAAT -3'/5'- CAAGGTGCCATATTCCATCAAG -3'.

Gbrγ2: 5'- TGGAATGATGGCAGAGTGTTG -3'/5'- GTAGTCTTCACCACCTCAGTTG -3'.

Gbrγ3: 5'- TTGCTGCTCTGATGGAGTATG -3'/5'- ACTGTAGTTGGTGGTCTCATATC -3'.

Gbrγ4: 5'-CAGAGATAGAGGAGGATGAAGATG-3'/5'- GAAGAGCAGGAAGGCAGTG -3'

β-tubulin: 5'- ATTGTGATGGACTCTGGT -3'/5'- TCGGCTGTGGTGGTGAAG -3'.

Expression levels were normalized to that of β –tubulin. Relative expression data was quantified using 2^-(Ct Control-Ct sample)^ where C_t_ is the cycle threshold. Relative standard curves were generated by plotting the threshold value (C_t_) versus the log of the amount of total cDNA added to the reaction and used to check the efficiency of primers. Calculation of C_t_, standard curve preparation and quantification of mRNA in the samples were performed by software provided with the Chromo4 (Opticon 3). All target genes were normalized to the β -tubulin housekeeping gene.

***Statistical analysis***

To determine significance, all data were subjected to statistical analysis using a computer program (GraphPad Prism). One-way ANOVA was used, as indicated in the figure legends, followed by a *post-hoc* test (Tukey) of differences between specific time points. All data are presented as the mean±SEM A level of *P*< 0.05 was considered significant.

## Results

GABA_A_ receptor gamma subunit genes showed significant alterations in chicken embryonic development. Figure 1 shows the temporal changes in mRNA expression of these genes. In each case, the data were expressed as a ratio to that expressed in 32 hr after hatching. 

***Temporal alterations in GABA***_A_ ***gamma 1 expression during development***

GABA_A_ gamma 1 expression was altered during embryonic development (Figure 1, panels A). One-way ANOVA indicated significant enhancment in gene expression during development. GABA_A_ gamma 1 mRNA was increased by 62.73±3.16 fold at 72 hr and 124.47±6.32, 438.01±22.65, and 1328.51±82.53 fold on 7, 14, and 20 days during development, respectively, then decreased by day one after hatching (518.85±44.22). Therefore, the pattern of GABA_A_ gamma 1 mRNA increase resembled a wave travelling during development.

***Temporal alterations in GABA***_A_ ***gamma 2 expression during development***

Different pattern was seen for the expression of GABA_A_ gamma 2 (Figure 1, panels B). GABA_A_ gamma 2 mRNA was significantly (*P*< 0.05) increased in all time points, and decreased in day one after hatching. The peak in enhancment was similar to the GABA_A_ gamma 1. 

***Temporal alterations in GABA***_A_ ***gamma 3 expression during development***

GABA_A_ gamma 3 mRNA (Figure 1, panels C) was higher than 32 hr on all days. There was a profound increase on 3 days followed by an increase on day 7, 14, and 20. The greatest alterations were seen on 20th day again. The mRNA expression level of GABA_A_ gamma 3 remained stable after hatching. 

***Temporal alterations in*** ***GABA***_A_ ***gamma 4*** ***expression during development***

GABA_A_ gamma 4 mRNA began to increase on day 14 (824.85±86.51) during development, and then continued to increase to 5680.22±542.02 fold on 20th day, and reduced to 4513.52±447.85 fold on day one after hatching. Enhancement on 20th day was significant (*P*< 0.00) (Figure 1, panel D).

## Discussion

Although GABAergic neurons and also a high amount of GABA in embryonic neurons and CNS of newborns have been found, the GABA may have different role in embryonic stages and mature ages (13). Regarding this, physiological and pharmacological features of GABA receptors may be changed during brain development. Northern and western blot analysis proved that age dependence occurs within α1, α2, α3, α5 and β1 subunits but the details in different brain zones have not been displayed. It has been reported that the expression of each GABA subunit receptor gene changes during the initial development (14). 

Rodriguez *et al* used "*in situ* hybridization" technique to analyze the site expression of four mRNAs, α1, α2, β2 and γ2 GABA receptor subunits, in the vision of developing chicken (9). 

All subunits that had already been expressed during the E10 level in most of the vision layers such as α1, α2, and β3 undergo remarkable changes from E10 to E20 level though γ1 undergoes just a few changes in some levels (9). The results of this experiment show that chicken brain have a low amount of γ1 subunit expression in the first days after birthand also a very low expression of γ4 in ED18. The results of this study demonstrate that neurons in different layers, show different expression paterns; influenced by many cellular treatments such as reproduction, after mitotic neuronic migration, cell programmed death, and differentiation, hense understanding this pattern becomes more complicated (9).

In the present study, the expression level of γ2, γ3 and γ4 started to increase on day 14 of embryonic develeopment and reached to its maximum through developmental embryonic stages.

Enomoto and colleagues measured the developmental changes of the expression of seven types of GABA receptor subunits (α1, β2, β3, β4, γ1, γ2 and γ4) in brainstem of chicken embryo during 2-20 days of incubation and also immediately after emerging from egg. The expression stage had been determined using RT-PCR. The electrophoresis patterns of γ4. Γ1, β3 and α1 RT-PCR products show that γ4 had a later arrival. The expression of α1, β3 and , γ1 has started during E2 to E6 levels but the expression of γ4 started in E8 and the expression increases during the development and arrives to a uniform step in E14. In brainstem between E4 to E7 just an isolated γ2 band was discovered although after E8 another band with a higher molecular weight appeared. In brain-stem, the γ2s or the smaler part, appeared in E4. The γ21, the larger part, at first appeard in E9 and got to a uniform step in E15. The absence of γ4 and γ21 in embriyonic stage shows the pharmacological difference of this subunits during development (11).

To date, however, very few investigations about the GABA receptors expression quantity in chicken embryo have been reported. In the present investigation, the quantitative expression of γ receptor subunits was done for the first time. 

Since the GABA receptors play a very important role in the nerveus system, functions of these receptors during the developmental phases are very important. Therefore, by knowing the expression changes of these receptores we can understand not only their physiological role but also their pharmacological features. 

In accordance to the report by Emoto and colleagues, γ1 started to increase at E4 stage and got to its maximum in E20 stage and decreased after emerging from egg. Moreover, the expression of γ2, γ3, γ3, and γ4 increased in E14 and reached to the maximum at the last stage. 

## Conclusions

All together, these data showed a spatiotemporal expression pattern for GABA_A_ receptor gamma subunits in forebrain that can explain the diversity of GABA_A_ receptor properties. 
